# Role of the NF-kB/parkin/vegfr-1 pathway associated with hypoxic-ischemic insult in germinal matrix samples of newborn infants

**DOI:** 10.1590/1984-0462/2023/41/2022034

**Published:** 2023-04-28

**Authors:** Eliane Amaral Ghirelli, Felipe Paes Gomes da Silva, Alessandro Gonçalves Gomes Oricil, Caroline Busatta Vaz de Paula, Seigo Nagashima, Carlos Frederico Oldenburg, Eduardo Storti, Fernando Yochiteru Rolim Sakiyama, Rafael Martins Kayano, Renata Rolim Sakiyama, Vinícius da Silva Moreira, Vanessa Santos Sotomaior, Lucia de Noronha

**Affiliations:** a Pontifícia Universidade Católica do Paraná, Curitiba, PR, Brazil.; b Universidade Federal do Paraná, Curitiba, PR, Brazil.

**Keywords:** Germinal matrix, NF-kB, VEGFR-1, Prematurity, Hypoxic-ischemic injury, Matriz germinativa, NF-kB, VEGFR-1, Prematuridade, Lesão hipóxico-isquêmica

## Abstract

**Objective:**

Given the high proliferative activity of germinal matrix and its direct correlation with hypoxemia, it is necessary to investigate the possible molecular regulation pathways, to understand the existing clinical relationship between the hypoxic-ischemic insult and the biomarkers NF-kB, AKT-3, Parkin, TRK-C and VEGFR-1.

**Methods:**

A hundred and eighteen germinal matrix samples of the central nervous system of patients who died in the first 28 days of life were submitted to histological and immunohistochemistry analysis to identify the tissue immunoexpression of those biomarkers related to asphyxia, prematurity, and death events within 24h.

**Results:**

A significantly increased tissue immunoexpression of NF-kB, AKT-3 and Parkin was observed in the germinal matrix of preterm infants. In addition, significantly decreased tissue immunoexpression of VEGFR-1 and NF-kB was observed in patients who experienced asphyxia followed by death within 24 hours.

**Conclusions:**

The results suggest a direct involvement between the hypoxic-ischemic insult and NF-kB and VEGFR-1 markers since a decreased immunoexpression of these biomarkers was observed in asphyxiated patients. Furthermore, it is suggested that there was not enough time for VEGFR-1 to be transcribed, translated and expressed on the surface of the plasma membrane. This temporality can be observed in the relationship between NF-kB expression and the survival time of individuals who died within 24 hours, suggesting that this factor is essential for the production of VEGFR-1 and, therefore, to carry out the necessary remodeling effect to neovascularize the affected region.

## RESUMO

**Objetivo:**

Dada a alta atividade proliferativa da matriz germinativa e sua correlação direta com a hipoxemia, é necessário investigar as possíveis vias de regulação molecular para entender a relação clínica existente entre o insulto hipóxico-isquêmico e os biomarcadores NF-kB, AKT -3, Parkina, TRK-C e VEGFR-1.

**Métodos:**

Cento e dezoito amostras de matriz germinativa do sistema nervoso central de pacientes que faleceram nos primeiros 28 dias de vida foram submetidas a análise histológica e imuno-histoquímica para identificar a imunoexpressão tecidual desses biomarcadores relacionados a eventos de asfixia, prematuridade e óbito em 24 horas.

**Resultados:**

Observou-se uma imunoexpressão tecidual significativamente aumentada de NF-kB, AKT-3 e Parkin na matriz germinativa de prematuros. Além disso, constatou-se uma imunoexpressão tecidual significativamente diminuída de VEGFR-1 e de NF-kB em pacientes que apresentaram asfixia seguida de morte em 24 horas.

**Conclusões:**

Os resultados sugerem o envolvimento direto entre o insulto hipóxico-isquêmico e os marcadores NF-kB e VEGFR-1, visto que se observou uma imunoexpressão diminuída destes biomarcadores nos pacientes asfixiados. Além disso, sugere-se que não houve tempo suficiente para que o VEGFR-1 fosse transcrito, traduzido e expresso na superfície da membrana plasmática. Essa temporalidade pode ser observada na relação entre a expressão de NF-kB e o tempo de vida dos indivíduos que morreram em 24 horas, o que sugere que esse fator é essencial para a produção do VEGFR-1 e, portanto, para realizar o efeito remodelador necessário para neovascularizar a região afetada.

## INTRODUCTION

According to the World Health Organization, 2.6 million newborns die in the first 28 days of life every year — and 75% of these deaths could be avoided. In Brazil, since the 1990s, neonatal death has been the main component of infant mortality^1,2^. The leading causes of neonatal death are congenital malformation, maternal factors, low birth weight, perinatal infections, prematurity, and respiratory injury, ranging from neonatal hypoxia to perinatal asphyxia (PA)^3,4^. Among the respiratory problems, PA is responsible for 23% of neonatal deaths^3^, which can be diagnosed based on blood gas analysis and Apgar score. In the fifth minute of the newborn’s life, the Apgar score becomes an essential tool for neuronal assessment^1^ — considering that asphyxia during childbirth can produce long-term deficits in brain development^5^.

The embryological development of the central nervous system (CNS) starts from the third week of fetal life, which is a dynamic and complex process that involves stages of differentiation, proliferation and neuronal migration — depending on the signaling pathway involved^6^. From the 12th week onwards, the Germinal Matrix (GM) formation begins — a thick layer with intense metabolism of subependymal cells of the thalamus-striatal sulcus. GM is richly vascularized, and its thin endothelial predisposes to a greater vulnerability of hemorrhagic affections until the 36th week, when it regresses^7,8,9^.

Regarding the differentiation that occurs in GM, after the closure process of the neural plate, the neuroepithelial cells (NEC) begin to proliferate and populate the periventricular zone, where the transition from NEC to radial glial progenitors (PGR) can occur^10^. The signaling pathways involved in this process are mediated by a family of molecules called neurotrophins. Activation through these molecules leads to autophosphorylation of tyrosine kinases (TRK) receptors, which will activate neuronal growth pathways, such as phosphatidylinositol 3-kinase/protein kinase B (PI3K/AKT), and therefore, will regulate the cell growth and proliferation^11^.

The AKT enzyme comprises a family of serine/threonine kinase proteins responsible for the phosphorylation of eukaryotic translation factors necessary for expressing genes related to cell survival^12^. Studies with knockout mice for AKT-3 have shown that a higher expression of this enzyme correlates to brain volume and better cognitive development in the animals tested^13^. In addition, this protein can positively regulate nuclear factor kappa B (NF-kB), promoting survival in response to apoptosis^12^.

NF-kB represents a protein complex belonging to a family of dimeric transcription factors formed by five prominent members. The regulation of the signaling pathways involved with the activation of NF-kB has a particular importance, given its close relationship with Parkin (a ubiquitin ligase E3 protein) and inflammatory factors released in a hypoxic-ischemic insult, such as tumor necrosis factor-alpha (TNF-α)^14^. In addition, NF-kB can promote the expression of genes involved in cell death and essential genes that act in cell survival and plasticity, such as the hypoxia-induced factor-1 (HIF-1)^15,16^.

HIF-1 is a heterodimer protein related to cell metabolism development, survival, and regulation. In studies performed with knockout mice for the HIF-1α gene, this protein has been proven to play an essential role in hypoxic-ischemic insults, mainly on germinal cells during brain development. During events of unbalanced cell oxygenation rates, the activation of the HIF-1 factor promotes the expression of receptors and neurotrophins involved in neovascularization, such as the vascular endothelial growth factor receptor-1 (VEGFR-1)^16,17^.

Considering GM’s high proliferative activity and immature CNS susceptibility to hypoxic-ischemic insults, a better investigation concerning the molecular pathways involved in the brain tissue response is required. By correlating some newborns’ clinical aspects with the biomarkers mentioned above, we may prove a sophisticated understanding of these proteins’ role in the developing CNS responses during a hypoxic-ischemic insult. This study investigates the relationship between the clinical signs of risk of CNS hypoxic-ischemic insult and the tissue expression of NF-kB, AKT-3, Parkin, TRK-C, and VEGFR-1 in GM of premature and term newborns, with or without clinical signs of neonatal hypoxia/asphyxia.

## METHOD

The present study was submitted and approved by the Research Ethics Committee of the Federal University of Paraná Hospital, in Curitiba, under the registration number: 1099.138/2005. The methodology was carried out following relevant guidelines and regulations. The patient’s legal representatives signed the Informed Consent Form (ICF).

The formalin-fixed paraffin-embedded (FFPE) GM fragments of the CNS of 118 patients who died (between 1991 and 2007) in the first 28 days of life (newborn) had their origin from the bank of necropsies of the Hospital de Clínicas (Curitiba, Brazil).

This sample was divided into two groups related to CNS immaturity (extremally immature CNS and non-immature CNS). Extremally immature CNS samples are those in which the gestational age was less than or equal to 30 weeks completed, while non-immature CNS samples comprised infants with gestational age over than or equal to 31 weeks. The newborns were evaluated for survival time in hours (time elapsed between birth and death) — patients who died up to 24 hours, between 25–72 hours, and over 72 hours. Additionally, they were evaluated for the presence or absence of asphyxia — patients with the Apgar score in the fifth minute lower than or equal to three were considered asphyxiated, and scores greater than three were considered not asphyxiated. The newborns were divided into three groups related to gestational age in weeks — less than or equal to 30 weeks completed, between 31 and 36 weeks completed, and over than or equal to 37 weeks), and birth weight in grams (less than or equal to 1000g, between 1,000 and 1,500g, and over than or equal to 1500g). Acidemia was defined by blood pH (first arterial blood gas to birth time) — less than or equal to 7.2. Low birth weight newborns (<P10) were also identified by correlating gestational age and birth weight — small for gestational age (SGA).

Other data such as gender, need for resuscitation in the delivery room, presence of associated malformations, presence of metabolic or mixed acidosis after birth, the underlying cause of death and other clinical conditions or associated diseases, as well as the presence or absence of risk factors for asphyxia, were also obtained through a direct search in the autopsy reports.

The histopathological findings were reviewed on slides stained with Hematoxylin and Eosin H&E (Harris Hematoxylin: NewProv, Cod. PA203, Paraná, BR; Eosin: BIOTEC Analytical Reagents, Cod. 4371, Paraná, BR).

Table 1 shows that the immunohistochemistry technique was applied to identify the tissue immunoexpression of NF-kB, AKT-3, Parkin, TRK-C, and VEGFR-1.

**Table 1.** Data of antibodies used in the immunohistochemistry technique to identify the tissue immunoexpression of biomarkers.

Antibody	Type	Clone/Code	Dilution	Source	Species reactivity
Anti-AKT-3	Monoclonal/Mouse	66C1247.1	1:400	Thermo Fisher	Human, Rat, Mouse
Anti-NF-kB p100/52	Polyclonal/Rabbit	ab7972	1:400	Abcam	Human, Rat, Mouse
Anti-PARK 2	Monoclonal/Mouse	PRK8	1:100	Abcam	Human, Rat, Mouse
Anti-TRK-C	Monoclonal/Mouse	75219	1:200	Leinco	Human
Anti-VEGFR-1	Polyclonal/Rabbit	PA1-21731	1:50	Thermo Fisher	Human, Rat, Mouse

The immunohistochemistry assay was preceded by making multi-sample paraffin tissue blocks, TM A (Tissue Microarray). The representative areas of the GM were previously demarcated and identified on the H&E slides, then cylindrical fragments from the GM areas, measuring approximately 0.3 cm in diameter, were extracted from the original blocks (donor blocks) and compiled into new TMA blocks.

The immunohistochemistry technique recommends an overnight incubation protocol for primary antibodies in a humid chamber at a temperature between 2 and 8°C. The secondary polymer (Reveal Polyvalent HRP-DAB Detection System, Spring Bioscience, Pleasanton, CA) was applied to the material tested for 20 minutes at room temperature. The technique was revealed by adding the 2, 3, diamino-benzidine complex + hydrogen peroxide substrate, for a brown color turning time, and later counterstaining with Harris Hematoxylin was performed. The results were confirmed by the reactivity of positive control (tissue sample with known positive immunoexpression for the antibody was allocated together with the samples studied).

Immunostained slides with NF-kB, AKT-3, Parkin, TRK-C, and VEGFR-1 were scanned by Axio Scan.Z1 slide scanner (Zeiss, Jena, Germany). The files generated from the digitization process were used to acquire 20 images in a medium-magnification field (MMF=20X objective) using the ZEN Blue Edition software (Zeiss, Jena, Germany). Analysis was performed blindly, so the generated images were obtained from random regions without the interference of the observer. The analysis of tissue immunoexpression areas was performed with Image Pro-Plus software version 4.5 (Media Cybernetics, Rockville, MD).

For each MMF, the tissue immunoexpression areas were obtained using a semi-automated color segmentation method, in which the immunopositive areas of the biomarkers were artificially delimited and quantified. Subsequently, the square micrometers (μm^2^) immunopositive values were converted into percentages (%). Afterwards, the mean values of the twenty MMF of each patient were calculated, and the results obtained were submitted to statistical analysis.

The qualitative variables of the groups defined by gestational age were compared using the chi-square test. Quantitative variables were analyzed using one-way analysis of variance (ANOVA) and Bonferroni’s post hoc test, or the non-parametric Kruskal-Wallis method. The Student’s t-test was used for independent samples and the non-parametric Mann-Whitney test to compare two groups regarding quantitative variables. The condition of normality of the variables was assessed using the Kolmogorov-Smirnov test. Quantitative variables that did not meet this condition had the data submitted to a logarithmic transformation. Values of p<0.05 indicated statistical significance. Data were analyzed using the computer program Stata/SE v.14.1. StataCorp LP, USA.

## RESULTS

Table 2 shows the clinical characteristics of patients such as gender, birth weight (grams), small gestation age (<P10), gestational age (weeks), CNS immaturity (gestational age≤30), acidemia (pH≤7,2), asphyxia (Apgar≤3) and survival time (hours). The present study is predominantly of male samples, totaling 66 patients. The prevailed weight was ≥1500g, observed in 55 patients (46.6%). The sample comprises 56 patients (47.5%) with gestational age ≤30 weeks and is mainly composed of non-SGA patients (101; 85.6%). Of the total cases, 79 (66.9%) presented acidemia, and 84 (71.2%s) did not present asphyxia. The predominant survival time was 25–72 hours for 48 patients (40.7%).

**Table 2.** Demographic characteristics and clinical findings.

Data	Category	Number of patients (%)	Mean±standard deviation
Gender	Male	66 (55.9)	--
	Female	52 (44.1)	
Birth weight (g)	≤1000	34 (28.8)	171±975
	1001-1499	29 (24.6)	
	≥1500	55 (46.6)	
Gestational age (weeks)	≤30	56 (47.5)	--
	31 a 36	36 (30.5)	
	≥37	26 (22.0)	
Small for gestational age (SGA)	No	101 (85.6)	--
	Yes	17 (14.4)	
Acidemia	No	39 (33.1)	--
	Yes	79 (66.9)	
Asphyxia (apgar score in the fifth minute ≤3)	No	84 (71.2)	5.6±2.8
	Yes	34 (28.8)	
Survival time (hours)	Until 24h	38 (32.2)	4.4±8.0
	25–72h	48 (40.7)	
	More than 72	32 (27.1)	

**Figure 1.**
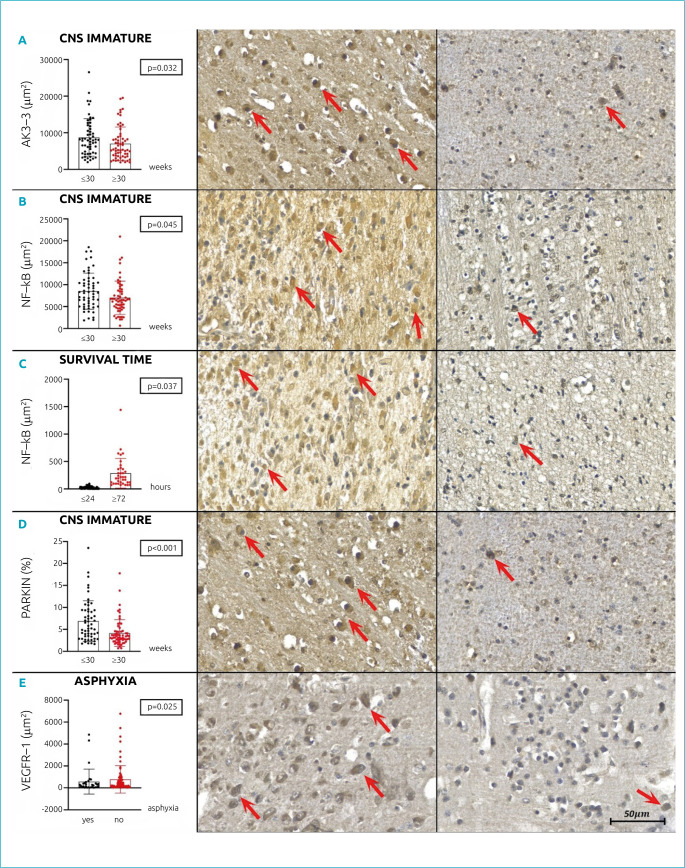
Graphics show tissue expression of Parkin, AKT-3, VEGFR-1, and NF-kB in columns plot mean±SD. Photomicrography shows germinal matrix expressing the respective biomarker — higher expression on the left column and lower expression on the right column (200X magnification).

The biomarkers studied and their correlation with clinical data are listed in Table 3. The AKT-3 tissue immunoexpression results showed higher values in newborns with extremally CNS immaturity (p=0.032; Figure 1A). Regarding NF-kB tissue immunoexpression, higher results were observed for newborns with extremally CNS immaturity (p=0.045; Figure 1B) and who survived over 72 hours (p=0.037; Figure 1C). As for Parkin, there was a significantly increased in tissue immunoexpression values in newborns with less than 1000g (p<0.001; Figure 1D), less than 30 weeks (p<0.001), with low birth weight for gestational age (p=0.014) and with extremal CNS immaturity (p<0.001; Figure 1A). The VEGFR-1 tissue immunoexpression showed higher values in newborns without asphyxia (p=0.025; Figure 1E).

**Table 3.** Biomarkers and their correlation with clinical data.

	Gender	p-value	Weight (g)	p-value	GA (weeks)	p-value	SGA	p-value	CNS (IG ≤30)	p-value	Acidemia (pH≤7,2)	p-value	Asphyxia (Apgar ≤3)	p-value	Survival (hours)	p-value
AKT-3	Male 8727±5578	0.094*	<1000 7483±3067	0.089*	≤30 8713±5119	0.089*	No 7956±4978	0.451*	No 7001±4550	0.032*	No 8612±6162	0.662*	No 7538±4864	0.300*	Until 24h 7202±4515	0.666*
			1000 a <1500 9778±6714		31 a 36 7302±4811										25–72h 8116±5228	
	Female 6641±3503		≥1500 7033±4504		≥37 6609±4245		Yes 6967±4280		Yes 8713±5119		Yes 7446±4122		Yes 8526±4948		Over 72h 8157±4889	
NF-Kb	Male 7401±3974	0.342*	<1000 8536±435	0.054*	≤30 8521±4112	0.092*	No 8000±4154	0.194*	No 7023±3787	0.045*	No 7707±4588	0.735*	No 7970±4126	0.402*	Until 24h 6374±3200	0.037**
			1000 a <1500 8329±3313		31 a 36 7381±3858										25–72h 8064±3958	
	Female 8195±4034		≥1500 6920±4025		≥37 6572±3720		Yes 6103±2243		Yes 8521±4112		Yes 7775±3725		Yes 7775±3725		Over 72h 8899 ± 4529	
Parkin	Male 570±440	0.726*	<1000 750±420	<0.001*	≤ 30 690±460	<0.001*	No 510±400	0.014*	No 420±300	<0.001*	No 630±430	0.355*	No 520±380	0.179*	Until 24h 550±420	0.988*
			1000 a <1500 600±450		31 a 36 490±350										25–72h 510±340	
	Female 510±360		≥1500 390±310		≥ 37 310±190		Yes 730±410		Yes 690±460		Yes 510±390		Yes 610±460		Over 72h 490±490	
TRK-C	Male 870±560	0.179	<1000 1090±68	0.073*	≤30 1040±680	0.137*	No 910±600	0.266*	No 850±510	0.098*	No 1050±720	0.197*	No 960±600	0.481*	Until 24h 870±570	0.545*
			1000 a <1500 1000±660		31 a 36 920±520										25–72h 1010±63	
	Female 1020±650		≥1500 800±490		≥37 740±470		Yes 1090±570		Yes 1040±680		Yes 880±530		Yes 870±610		Over 72h 900±590	
VEGFR1	Male 629±1021	0.221*	<1000 881±1427	0.668*	≤ 30 908±1397	0.528*	No 648±1014	0.637*	No 551±1010	0.286*	No 899±1625	0.928*	No 778±1247	0.025*	Until 24h 697±1343	0.180*
			1000 a <1500 894±1312		31 a 36 574±1252										25–72h 941±1418	
	Female 823±1412		≥1500 526±996		≥37 521±576		Yes 1125±2026		Yes 908±1397		Yes 632±963		Yes 572±1137		Over 72h 387±293	

GA: gestational age; SGA: small for gestational age; CNS: central nervous system. *p obtained by Student’s t-teste. Bold indicates statistically significant p-values.

## DISCUSSION

During the GM proliferation phase, which lasts until the 36th week of pregnancy, there is an intense activation of TRK, which permeates the direct activation of the cascade regulated by the AKT-3 enzyme^11^, indispensable for the positive signaling of eukaryotic initiating factors eIF4-E and eIF4-B — which are necessary to produce proteins responsible for the formation of the cytoskeleton of mature neural cells.

The intense proliferative activity of GM associated with de higher tissue expression of AKT-3 can be observed in post-mortem tissue samples from the extremally immature CNS group (p=0.034), suggesting the need for high kinase levels to support the intense proliferative activity of the GM in developing brains.

Parkin is another crucial molecule in the developing brains and showed higher tissue expression in newborns with extremal CNS immaturity (p<0.001). This ubiquitin ligase E3 was also higher (p<0.001) in newborns with lower gestational age (≤30 weeks), lower birth weight (<1,000g), and SGA <P10, given its role in the degradation of defective proteins produced during the most intense neural development in GM. Thus, during this phase, Parkin would be protecting this immature brain against possible injuries^14^.

In addition to its proliferative activity, GM has an essential role in cell survival and defense against metabolic and/or infectious insults once it is already populated by glial cells, differentiated into a defense lineage, such as microglia. These cells can release pro-inflammatory molecules during insults to activate neuronal remodeling pathways necessary for survival^18,19,20^.

The presence of microglia was confirmed in the present study, with the immunostaining of anti-CD 68, suggesting that GM has these specialized cells for defense, which can release pro-inflammatory agents such as TNF-α, acting as neuromodulators of the surrounding cells. Therefore, this study compared tissue expression of Parkin, NF-kB, and VEGFR-1 in newborns with different birth weights, gestational ages, and degrees of CNS injury, to identify the remodeling pathway activated during the hypoxic-ischemic insult.

The neovascularization event can occur during the brain’s normal development or stressful events, such as O2 deprivation in the hypoxic-ischemic insult^17^. When this deprivation occurs, CNS cells induce factors such as VEGF and its receptor VEGFR-1, responsible for angiogenesis, to allow the affected region to receive oxygen again^16^. So, during this event, the expression of VEGFR-1 should be increased in injured newborns. Therefore, we observed lower tissue expression of VEGFR-1 in asphyxiated individuals (p=0.025).

Low levels of VEGFR-1 may be due to the cell time-producing or immaturity of the neuron cells. It is suggested that there was not enough time for the receptor to be transcribed, translated, and, therefore, expressed on the surface of the cell membrane. Therefore, even if produced by the AKT-3/mTOR pathways, the neurotrophin VEGF would not have its receptor for binding. The present study analyzed the tissue expression of its precursor molecule, the nuclear factor kappa B (NF-kB), trying to explain the cell time-producing of VEGFR-1 or the immaturity of neuron cells.

When analyzing the expression of the NF-kB factor according to the time elapsed between birth and death and immaturity of CNS, there was a reduction in the tissue expression (p=0.037) in those newborns who died within 24 hours and those who had extremally immaturity of CNS (p=0.045). The lower tissue expression of NF-kB in these groups of newborns could trigger a lower transcription of the HIF-1α, a precursor of VEGFR-1. Thus, the GM seems unable to perform the necessary remodeling effect to neovascularize the affected region, worsening the hypoxic-ischemic insult.

The most important limitation of our study that merits consideration is that data based on FFPE *post-mortem* samples only provide static information at the time of death, which does not allow the reconstruction of the evolving disease process.

In conclusion, this study hypothesized the pathway illustrated in Figure 2.14,16,18,20-25 This view of molecular signaling mechanism, evidenced through the immunoexpression of the markers (studied in the ischemic process of the germinal matrix), suggests the development of future work in therapeutic field: the reduction of neural damage caused by respiratory disease in neonates by inducing neural remodeling and, thus, GM neovascularization.

**Figure 2.**
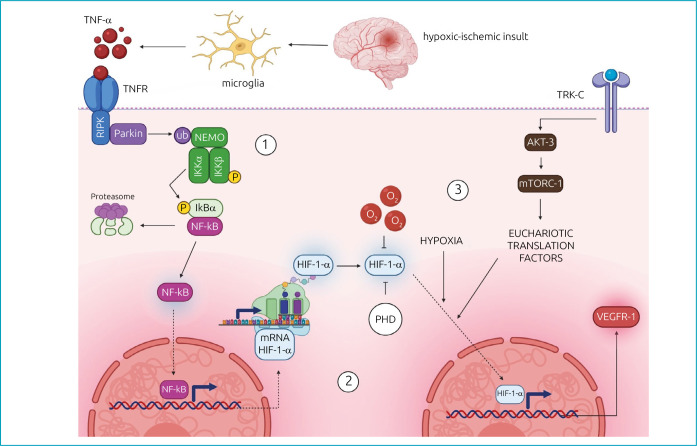
(1) Recruitment of Parkin by inflammatory agents in the activation process of nuclear factor-kappa B (NF-kB): In the course of the hypoxic-ischemic insult, inflammatory factors are released by glial cells, such as TNF-α, activating a pathway of neural remodeling^18,20^. In the presence of high levels of TNF-α, it binds to its receptor, TNFR, which recruits the protein kinase interaction receptor (RIPK), which serves as a binding site for Parkin to act. This molecule promotes the activation of nuclear factor-kappa (NF-kB) by promoting the ubiquitination of the gamma (NEMO) subunit of the IkB enzyme complex (IKK)^14^. Thus, Parkin promotes NEMO labeling and indirectly phosphorylation of the Beta subunit (IKKβ) of the IKK complex by an upstream kinase, activating it^21,22^. With this activation, IKKβ manages to phosphorylate the inhibitory protein linked to NF-kB, IkBα, which is polyubitkinated and degraded by proteosomes, thus allowing NF-kB, originating from the canonical pathway, to enter the nucleus of neuronal cells in its active form. (2) Role of NF-kB in HIF-1-α mRNA transcription: The NF-kB activated binds to the initiation site of the HIF-1-α precursor DNA molecule, which induces the transcription of the mRNA HIF-1-α^23^.This binding allows translation of the HIF factor promoter gene, giving rise to the HIF-1-α molecule. This molecule, inside the cell cytoplasm and in the presence of oxygen, is induced to degradation by prolyl hydroxylase (PHD) enzymes and, under hypoxic conditions, allows the HIF molecule to be activated and enter the cell nucleus^16^. (3) Role of cell growth pathways in HIF activation and production of neural protective factors in MG such as VEGFR-1: Once the HIF molecule is produced, it needs to be stabilized and activated in order to be able to promote the production of neural protective factors such as VEGFR-1. The HIF1-α molecule in the cell cytoplasm is inactive and stabilized by normoxic conditions. For its activation to occur, several factors are related, among them, eukaryotic translation factors/growth factors, which have been shown to contribute to HIF-1-α activation by phosphorylating the molecule and allowing it to enter the nucleus^24,25^. In this process, neurotrophins are released and bind to the Tyrosine Kinase C (TRK-C) receptor, which is responsible for activating the AKT-3/mTORC-1 pathway and producing eukaryotic translation factors. Inside the nucleus, HIF can produce molecules associated with cellular vascularization — VEGFR-1 — and, therefore, revitalize the tissue by guaranteeing the supply of O2.

## Data Availability

The database that originated the article is available with the corresponding author.

## References

[1] Vargas NS (2012). Marcadores prognósticos de evolução neonatal de recém-nascidos de termo portadores de asfixia perinatal [dissertation].

[2] Veloso FC, Kassar LM, Oliveira MJ, Lima TH, Bueno NB, Gurgel RQ (2019). Analysis of neonatal mortality risk factors in Brazil: a systematic review and meta-analysis of observational studies. J Pediatr (Rio J).

[3] Lansky S, Friche AA, Silva AA, Campos D, Bittencourt SD, Carvalho ML (2014). Pesquisa nascer no Brasil: perfil da mortalidade neonatal e avaliação da assistência à gestante e ao recém-nascido. Cad Saúde Pública..

[4] Pedrosa LD, Sarinho SW, Ordonha MA (2005). Óbitos neonatais: por que e como informar?. Rev Bras Saude Matern Infant..

[5] Morales P, Bustamante D, Espina-Marchant P, Neira-Peña T, Gutiérrez-Hernández MA, Allende-Castro C (2011). Pathophysiology of perinatal asphyxia: can we predict and improve individual outcomes?. EPMAJ..

[6] Higginbotham H, Yokota Y, Anton ES (2011). Strategies for analyzing neuronal progenitor development and neuronal migration in the developing cerebral cortex. Cereb Cortex..

[7] Ballabh P, Vries LS (2021). White matter injury in infants with intraventricular haemorrhage: mechanisms and therapies. Nat Rev Neurol..

[8] Ballabh P, Braun A, Nedergaard M (2004). Anatomic analysis of blood vessels in germinal matrix, cerebral cortex, and white matter in developing infants. Pediatr Res..

[9] Raets MM, Dudink J, Govaert P (2015). Neonatal disorders of germinal matrix. J Matern Neonatal Med..

[10] Jossin Y (2004). Neuronal migration and the role of reelin during early development of the cerebral cortex. Mol Neurobiol..

[11] Kandel ER (2014). Príncípios de neurociências.

[12] Matsuo FS (2015). Estudo da via de sinalização PI3K-Akt e GSK3β em carcinomas epidermoides metastáticos e não metastáticos de cavidade bucal [dissertation].

[13] Osaki M, Oshimura M, Ito H (2004). PI3K-Akt pathway: its functions and alterations in human cancer. Apoptosis..

[14] Wang Y, Shan B, Liang Y, Wei H, Yuan J (2018). Parkin regulates NF-κB by mediating site-specific ubiquitination of RIPK1. Cell Death Dis..

[15] Franco DG (2010). Fator de transcrição nuclear kappa B no sistema nervoso central: do fisiológico ao patológico. Rev Biol..

[16] van Uden P, Kenneth NS, Rocha S (2008). Regulation of hypoxia-inducible factor-1alpha by NF-kappaB. Biochem J..

[17] Wenger RH (2002). Cellular adaptation to hypoxia: O2 -sensing protein hydroxylases, hypoxia-inducible transcription factors, and O2 -regulated gene expression. FASEB J..

[18] Kaur C, Hao AJ, Wu CH, Ling EA (2001). Origin of microglia. Microsc Res Tech..

[19] Santos NK (2013). Avaliação da inflamação por mensuração de IL-1β em modelo animal de hemorragia da matriz germinativa/intraventricular perinatal [TCC].

[20] Supramaniam V, Vontell R, Srinivasan L, Wyatt-Ashmead J, Hagberg H, Rutherford M (2013). Microglia activation in the extremely preterm human brain. Pediatr Res..

[21] Hayden MS, Ghosh S (2014). Regulation of NF-κB by TNF family cytokines. Semin Immunol..

[22] Mulero MC, Huxford T, Ghosh G (2019). NF-κB, IκB, and IKK: integral components of immune system signaling. Adv Exp Med Biol..

[23] Minet E, Ernest I, Michel G, Roland I, Remacle J, Raes M (1999). HIF1A gene transcription is dependent on a core promoter sequence encompassing activating and inhibiting sequences located upstream from the transcription initiation site and cis-elements located within the 5’UTR. Biochem Biophys Res Commun..

[24] Jiang BH, Zheng JZ, Aoki M, Vogt PK (2000). Phosphatidylinositol 3-kinase signaling mediates angiogenesis and expression of vascular endothelial growth factors in endothelial cells. Proc Natl Acad Sci U S A..

[25] Chen EY, Mazure NM, Cooper JA, Giaccia AJ (2001). Hypoxia activates a platelet-derived growth factor receptor/phosphatidylinositol 3-kinase/Akt pathway that results in glycogen synthase kinase-3 inactivation. Cancer Res..

